# Am80-Encapsulated Lipid Nanoparticles, Developed with the Aim of Achieving Alveolar Regeneration, Have an Improvement Effect on Pulmonary Emphysema

**DOI:** 10.3390/pharmaceutics15010037

**Published:** 2022-12-22

**Authors:** Tomomi Akita, Yuki Morita, Takehiro Kawai, Kazuaki Oda, Kota Tange, Yuta Nakai, Chikamasa Yamashita

**Affiliations:** 1 Department of Pharmaceutics and Drug Delivery, Faculty of Pharmaceutical Sciences, Tokyo University of Science, 2641 Yamazaki, Noda 278-8510, Japan; 2 DDS Research Laboratory, NOF CORPORATION, 3-3 Chidori-cho, Kawasaki-ku, Kawasaki City 210-0865, Japan

**Keywords:** chronic obstructive pulmonary disease, lipid nanoparticle, alveolar regeneration, Am80, pulmonary administration

## Abstract

Chronic obstructive pulmonary disease (COPD) is characterized by chronic bronchitis and emphysema, and current drug treatments target its symptoms. Thus, the development of a therapeutic drug to repair alveolar destruction is urgently needed. Our previous research revealed that the synthetic retinoic acid Am80 (1.0 mg/kg) showed a repairing effect on collapsed alveoli in a mouse model of elastase-induced emphysema. However, a further reduction in the dose is desirable to facilitate the development of a powder inhalation formulation for clinical application. We, therefore, focused on SS-OP to deliver Am80 efficiently. As a result, 0.01 mg/kg of Am80-encapsulated SS-OP nanoparticles repaired collapsed alveoli and improved the respiratory function in the mouse model of elastase induced emphysema. The results suggested that, with the use of SS-OP, the Am80 dose could be reduced. This could contribute to the development of a powder inhalation system as a curative medicine for COPD.

## 1. Introduction

Chronic obstructive pulmonary disease (COPD) is a lung disease caused by prolonged inhalation of toxic substances and patients with COPD show airflow obstruction on respiratory function tests. Airflow obstruction is usually progressive, involving a combination of small airway lesions (bronchiolitis obliterans) and emphysematous lesions (emphysema) in varying proportions [1]. Although smoking is the greatest risk factor, COPD actually occurs in approximately 15–20% of smokers [2]. Thus, it is considered that multiple genetic factors are involved in the onset of COPD, and that the onset of COPD is caused by the addition of environmental factors (e.g., smoking) to genetic factors [3]. In addition, although the protease–antiprotease imbalance theory and oxidant–antioxidant imbalance theory have long been proposed as the pathogenesis of COPD, the full extent of these theories has not yet been clarified [4,5]. In addition, in recent years, it has been proposed that the acceleration of apoptosis and cellular senescence is an important pathogenesis of COPD [4], and that persistent inflammation and serious damage to the respiratory tract and lungs in childhood lead to impaired lung growth in adulthood, increasing the risk of developing COPD [5]. Approximately 384 million people worldwide suffer from COPD [1], and more than half of COPD patients are undiagnosed [6]. Furthermore, according to a World Health Organization (WHO) study, COPD was the third leading cause of death worldwide in 2019 [7].

Although COPD is becoming a global disease, there are currently no drugs that can repair the alveolar destruction that occurs during the disease. A long-acting muscarinic antagonist (LAMA), inhaled steroids and long-acting β2 agonists (LABA) are used as symptomatic treatment [8]; however, treatment satisfaction among COPD patients remains at 60% [9]. From these reports, there is an urgent need to develop a radical treatment that can repair collapsed alveoli for COPD.

In recent years, regenerative medicine has become increasingly important for the repair of injured tissue. Regenerative medicine is a method of regenerating defective biological tissue by utilizing the proliferation and differentiation of cells to maximize the natural healing power of the organism. Studies on endogenous progenitor cells and differentiation inducers, in efforts to regenerate and treat lung damage, have attracted attention [10]. The existence of tissue progenitor cells was also suggested in the lung, and human alveolar epithelial progenitor cells were identified [11]. The alveoli terminal structures of the distal airways have a major role in gas exchange; their surfaces are occupied by type I and type II alveolar epithelial cells [12]. In COPD, the irreversible destruction of alveoli makes gas exchange difficult, so it is considered that the complete cure of COPD can be achieved by differentiating alveolar epithelial progenitor cells into type I or type II alveolar epithelial cells that constitute alveoli and regenerating alveoli. To date, all-*trans* retinoic acid (ATRA) has been reported to be an important signaling molecule for differentiation [13,14,15,16]. However, a significant therapeutic effect was not obtained in a human clinical study by the oral administration in COPD patients, and the cause is considered to be metabolism by CYP26 [17,18]. Then, we focused on a synthetic retinoid tamibarotene (Am80; (4- [(5, 6, 7, 8-Thetrahydro -5, 5, 8, 8-tetramethyl -2 naphthyl) carbamoyl] benzoic acid)), which is a selective agonist for nuclear retinoic acid receptor (RAR) α and β. Am80 was developed to improve therapeutic efficacy and reduce side-effects. It is reported that the differentiation induction potency of Am80 for HL-60 cells (Human promyelocytic leukemia cells) and NB-4 (Human leukemia) cells is 10 times stronger than that of ATRA [19]. As for the metabolic difference, Am80 is resistant to metabolism by CYP26 [20]. Our previous study reported that Am80 exerts alveolar repair effects in COPD model mice after pulmonary administration at a dose of 1.0 mg/kg [21]. Based on these results, the clinical dose calculated in accordance with FDA guidance is estimated to be 5.0 mg/60 kg [22]. A further dose reduction is desirable to facilitate the development of a powder inhalation formulation for clinical application.

As it was reported that the site of action of Am80 is the nuclear receptor RAR [23,24], we thought that the nuclear Am80 concentration would need to be increased in order to reduce the dose of Am80. For this purpose, efficient drug delivery into the cytoplasm is first considered necessary. Therefore, to efficiently deliver Am80, we focused on SS-cleavable proton-activated lipid-like material O-Phenyl-P4C2 (SS-OP), which was developed as a nucleic acid delivery carrier [25]. SS-OP has two intracellular environmental response units, “tertiary amines” and “disulfide bonds”. After internalization, SS-OP nanoparticles exhibit a proton sponge effect in which the surface of the particle is positively charged, disrupting and escaping the endosome by protonation of a tertiary amine in response to a decrease in pH as the endosome matures [26]. Furthermore, the surface is destabilized by the cleavage of the intramolecular disulfide bond by glutathione in the cytoplasm, and the drug is released by self-disintegration through the intraparticle reaction between the resulting thiol group and the intramolecular phenyl ester, so efficient drug delivery to the cytoplasm can be expected [25].

In this study, we investigated the usefulness of SS-OP nanoparticles containing Am80 for improving pulmonary emphysema, with the aim of reducing the dose of Am80 for the treatment of COPD.

## 2. Materials and Methods

### 2.1. Cell Culture

The undifferentiated human lung cancer cell line Calu-6 (ATCC, Manassas, VA, USA) was used. Calu-6 cells were cultured in Eagle’s minimal essential (EMEM) medium (Fujifilm Wako Pure Chemical Industries, Osaka, Japan) supplemented with 10% FBS, MEM non-essential amino acids (Fujifilm Wako Pure Chemical Industries, Osaka, Japan), 1 mM sodium pyruvate (Fujifilm Wako Pure Chemical Industries), and 0.1% penicillin-streptomycin (Life Technology Co., New York, NY, USA) under 5% CO_2_ at 37 °C.

### 2.2. Animal Species

Male ICR mice (age: 5 weeks) were purchased from Sankyo Labo Service Corporation, Inc. (Tokyo, Japan). Mice that were fed had ad libitum access to standard solid chow and sterilized water. After 1 week of acclimation in an SPF room with a 12 h light/dark cycle, the 6-week-old mice were used for the experiment. This experiment was conducted with the approval of the Tokyo University of Science Ethics Committee (approval number: Y20032).

### 2.3. Model Mouse Preparation and Drug Administration

**Figure 1:**
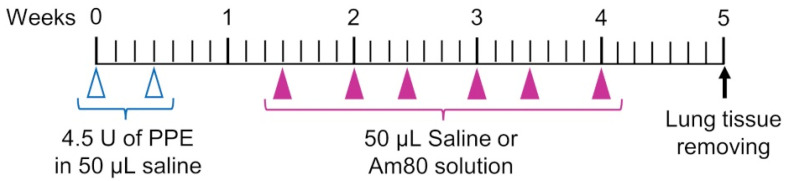
Experimental schedule.

The elastase-induced COPD model mouse used in this study can easily be produced, and elastase degrades elastin in the lung and causes a loss of elasticity in the lung. Among mouse models of COPD, this model is useful for evaluating local injury and effects in the lung [27,28,29]. The timeline for animal experiments is summarized in Figure 1. For the PPE dose and period of inducing emphysema, we referred to a previous study in which emphysema was already induced by 1 week after administration of PPE and used it with some modifications [21,30]. The PPE model used in this study is used as a model for treating diseases. Porcine pancreatic elastase (PPE) (Elastin Products Company, Owensville, MO, USA) was dissolved in saline (Otsuka Pharmaceutical Co., Tokyo, Japan), and a solution of PPE (4.5 units/50 μL of saline) was pulmonary-administered to 6-week-old male mice twice a week (3 days interval) for 1 week to create an elastase-induced COPD model mouse. From 1 week after the final administration of PPE, the pulmonary administration of Am80 (0.1 mg/kg and 1.0 mg/kg) solution and Am80 (0.01 mg/kg and 0.1 mg/kg)-encapsulated SS-OP nanoparticle suspension was performed at a dose of 50 μL/head twice a week for 3 weeks. The Control group received 50 μL/head of saline and the Placebo group received 50 µL/head of SS-OP nanoparticle suspension (pulmonary administration) without the inclusion of Am80. The timeline for administration of the drug solution was based on our previous research [31,32,33]. The Am80 solution was prepared with saline so that the dimethylsulfoxide (DMSO) (Fujifilm Wako Pure Chemical Industries, Osaka, Japan) concentration was 5% and the Tween 80 (Tokyo Chemical Industry, Tokyo, Japan) concentration was 0.05%.

The pulmonary administration used in this study was the negative-pressure method that we previously established [34]. It was carried out with a stainless-steel oral sonde for oral administration (N-PK 002, Nihon Bioresearch Inc., Nagoya, Japan), which is equivalent in diameter to the mouse airway. Mice were anesthetized with isoflurane. Using the Mouse Intubation Platform–Model MIP (Penn–Century, Inc., Wyndmoor, PA, USA) as a mouse retainer for pulmonary administration, the front teeth of the mouse were retained at approximately 90° in the retaining position to facilitate tracheal access of the oral sonde. The airway was confirmed with the Small Animal Laryngoscope–Model LS-2 (Penn–Century, Inc., Wyndmoor, PA, USA) as a tracheal endoscope for the mouse, the oral sonde was inserted into the airway, and the drug solution was administered with the negative pressure of inhalation generated by the mouse.

### 2.4. Nanoparticle Preparation

Am80 was provided by Dr. Koichi Shudo at the University of Tokyo and was dissolved in ethanol (Fujifilm Wako Pure Chemical Industries, Osaka, Japan) to make a 5 mM solution. SS-OP (COATSOME^®^ SS-OP), 1,2-Dioleoyl-sn-glycero-3-phosphoethanolamine (DOPE) (COATSOME^®^ ME-8181), and 1,2-Dimyristoyl-rac-glycero-3-methylpolyoxyethylen (DMG-PEG 2000) (SUNBRIGHT^®^ GM-020) were provided by NOF CORPORATION (Tokyo, Japan). Cholesterol was provided by TOKYO CHEMICAL INDUSTRY (Tokyo, Japan). SS-OP, DOPE, and cholesterol were dissolved in ethanol to make a 5 mM solution. DMG-PEG 2000 was dissolved in ethanol to make a 1 mM solution. The lipid ethanol solution was obtained by mixing 240 μL of SS-OP, 320 μL of DOPE, 240 μL of cholesterol, 400 μL of DMG-PEG 2000, and 400 μL of Am80. An amount of 20 mM malic acid/NaCl buffer (pH 3.0 with 1250 mM NaCl), lipid ethanol solution, and saline was ice cooled. The lipid ethanol solution was added with 1600 μL of 20 mM malic acid/NaCl buffer and 11 mL of saline with stirring. Thereafter, the mixture was transferred to an ultrafiltration unit (Amicon Ultra 15 filter 100 kDa, Merck Millipore Co., Burlington, MA, USA) and centrifuged at 3000 rpm at 26 °C to concentrate the particle suspension to approximately 2.5 mL. Then, 10 mL of saline was added and the solution was centrifuged again. This procedure was repeated three times to replace the solvent with saline. A final volume of 800 µL gave an SS-OP nanoparticle suspension with a lipid concentration of 5.5 mM.

### 2.5. Evaluation of Physical Properties and Measurement of the Encapsulation Rate

To evaluate the physical properties, the particle size and zeta potential of the prepared SS-OP nanoparticles were measured using an ELSZ-2000 (Otsuka Electronics Co., Ltd., Osaka, Japan). The concentration of Am80 encapsulated in the prepared Am80-encapsulated SS-OP nanoparticles was measured by a high-performance liquid chromatography (HPLC) Nexera X2 (Shimadzu, Kyoto, Japan) using an Inert Sustain C18 Column (4.6 × 250 mm, 5 µm) (GL Sciences, Tokyo, Japan). The mobile phase was composed of ethanol and acetonitrile, using gradient elution, and performed at 40 °C with a flow rate of 1.0 mL/min. The concentration gradient of the mobile phase was controlled as follows: 0 min, 5% acetonitrile; 10 min, 30% acetonitrile; 20–25 min, 95% acetonitrile; 28–40 min, 5% acetonitrile. The eluate was monitored by a UV detector at 285 nm. 

### 2.6. Differentiation Inducibility

After seeding Calu-6 cells on 8-well culture slides (SPL Life Sciences Co. Gyeonggi-do, Korea) at 4.0 × 10^3^ cells/well, the cells were incubated overnight in 500 μL/well of complete culture medium. Cells were treated with Am80 diluted to 10 and 100 μM and Am80-encapsulated SS-OP nanoparticles diluted to 0.1, 1, and 10 μM every 48 h. After 6 days of treatment, cells were fixed in 4% paraformaldehyde (PFA) in PBS for 15 min at room temperature, followed by 3 repeated washes with PBS for 5 min, and blocking with PBS containing 10% BSA. After the reaction, washing with PBS for 5 min was repeated 3 times. Thereafter, slides were filled with a solution of primary antibodies (goat anti-human Thy1 (CD90) antibody (sc-6071, Santa Cruz Biotechnology, Santa Cruz, CA, USA), mouse anti-human AQP-5 antibody (sc-514022, Santa Cruz Biotechnology, Santa Cruz, CA, USA), and goat anti-human SP-A antibody (sc-7699, Santa Cruz Biotechnology, Santa Cruz, CA, USA)), diluted 1:200 with PBS solution, and reacted overnight at 4 °C. After the reaction, cells were washed 3 times for 5 min with PBS containing 0.1% Tween 80 (PBST). Next, as the secondary antibody, donkey anti-goat IgG H&L (FITC) (Merck Millipore, Darmstadt, Germany) diluted 1:200 in PBS solution and 4′,6-diamidino-2-phenylindole (DAPI) (Roche Applied Sciences Co. Mannheim, Germany) for nuclear staining was filled with a mixed solution of 20 μg/mL and reacted at room temperature for 90 min. After the reaction, washing with PBST was performed 5 min and repeated 3 times. Then, the observation was performed with a fluorescence microscope BZ-9000 (Keyence, Osaka, Japan), and the number of positive cells with each unique marker was counted using the Image J software program (National Institutes of Health, Bethesda, MD, USA). The percentage of positive cells was calculated as the number of unique marker-positive cells/number of 500 DAPI-positive cells × 100.

### 2.7. Tissue Immunofluorescence Staining

One week after the administration of the drug, mice were euthanized by an overdose of isoflurane, and lung tissue was removed and fixed using 4% PFA in PBS. The fixed lung tissue was embedded using a Tissue-Tek O.C.T compound (Sakura Finetek Japan Co., Ltd., Tokyo, Japan). Thereafter, frozen sections were prepared to a thickness of 10 μm using a cryostat CM3050 (Leica Micro systems GmbH, Wetzlar, Germany). Then, the type I alveolar epithelial cell marker AQP-5 and type II alveolar epithelial cell marker SP-A were determined by immunofluorescence staining. The details of the staining method for AQP-5 are shown below. The samples were dried for 3 min, immersed in 100% ethanol for 5 min, fixed with 4% PFA in PBS for 15 min at room temperature, and re-fixed. After washing 2 times with 0.025% Triton-100/TBS for 5 min, blocking was performed using 1% BSA/TBS in 10% goat serum. After 120 min of reaction, lung tissue sections were exposed to a solution of primary antibody Rabbit anti-Aquaporin 5 antibody (Millipore, Billerica, MA, USA) diluted 1:100 in 1 × TBS and allowed to stand overnight at 4 °C. Thereafter, lung sections were soaked and washed 2 times with 0.025% Triton-100/TBS for 5 min and exposed to secondary antibody Alexa Fluor 594 conjugate anti-rabbit IgG (Cell signaling Technology Co., Danvers, MA, USA) and DAPI solution diluted 200-fold with 1 × TBS for 90 min at room temperature. Lung sections exposed to the secondary antibody were washed 3 times for 5 min with 1x TBS, and Cryofilm type 2C (9) (Section Lab Co., Ltd., Hiroshima, Japan) and lung sections were attached to slides using Fluoromount ^TM^ (Roche Applied Sciences Co., Mannheim, Germany). Lung sections were photographed with a BZ-9000 fluorescence microscope (Keyence, Osaka, Japan). The procedure for staining SP-A is the same as that for AQP-5, except for blocking. Lung sections were blocked using 1% BSA-containing PBS and allowed to react for 60 min, after which lung tissue sections were exposed to a solution of primary antibody Rabbit anti-human SP-A antibody (abcam, Cambridge, UK) diluted 1:200 in 1 × PBS.

### 2.8. Alveolar Repair Effect

One week after the administration of the drug, the mice were euthanized by an overdose of isoflurane, and lung tissue was removed and fixed using 4% PFA in PBS. The fixed lung tissue was embedded using the Tissue-Tek O.C.T. compound (Sakura Finetek Japan Co., Ltd.). Thereafter, frozen sections were prepared to a thickness of 10 μm using a cryostat CM3050 (Leica Micro systems GmbH), and hematoxylin–eosin staining (H&E) was performed. Cryofilm type 2C (9) and stained lung sections were attached to slides using Fluoromount™. Lung sections were photographed with a BZ-9000 fluorescence microscope. Then, in order to examine the progression of alveolar destruction, the mean linear intercept (Lm) (National Institutes of Health, MD, USA) was calculated from the following equation using ImageJ (National Institutes of Health, MD, USA):

Mean linear intercept (μm) = number of intersections of the number of grids present in the alveoli × length between the intersections (µm)/number of alveoli in the field.

### 2.9. Improvement of Respiratory Function

One week after the end of the administration of the drug, mice were given 100 μL of pentobarbital sodium salt (70 mg/kg; Nacalai Tesque, Kyoto, Japan) and xylazine hydrochloride (12 mg/kg; Wako Pure Chemical Industries, Osaka, Japan) by intraperitoneal injection, and the trachea was cannulated through an incision in the chest and secured with sterile thread. Then, the forced expiratory volume in 0.05 s (FEV0.05%) was measured using flexiVent (TMSCIREQ©, Montreal, QC, Canada) under the following measurement conditions: ventilator rate, 200/minute; tidal volume, 10 mL/kg; positive end-expiratory pressure, 3 cm H_2_O; pressure limit, 30 cm H_2_O.

### 2.10. Statistical Analysis

Data were expressed as the mean ± standard error (S.E.) or standard deviation (S.D.). Dunnett’s test was used to test the significance of difference between other groups. The significance level was set at 0.05 in all analyses.

## 3. Results

### 3.1. Evaluation of the Physical Properties and Differentiation Induction Potency of SS-OP Nanoparticles

#### 3.1.1. Evaluation of the Physical Properties of SS-OP Nanoparticles

The particle size, zeta potential, and encapsulation rate of Am80 were measured as physical properties of the prepared Am80-encapsulated SS-OP nanoparticle solution. The particle size and zeta potential were measured using an ELSZ-2000. The encapsulation rate of Am80 was calculated based on the results of HPLC. The prepared Am80-encapsulated SS-OP nanoparticles had an average particle size of 125.7 ± 2.40 nm, a zeta potential of −4.07 ± 1.54 mV, and an encapsulation rate of 71.3 ± 8.49%. On the other hand, placebo SS-OP nanoparticles without Am 80 had an average particle size of 126.3 ± 2.32 nm and a zeta potential of −8.63 ± 4.72 mV (Table 1). The particle size of each nanoparticle showed a uniform unimodal distribution. 

#### 3.1.2. Differentiation Induction Potency of SS-OP Nanoparticles

**Figure 2:**
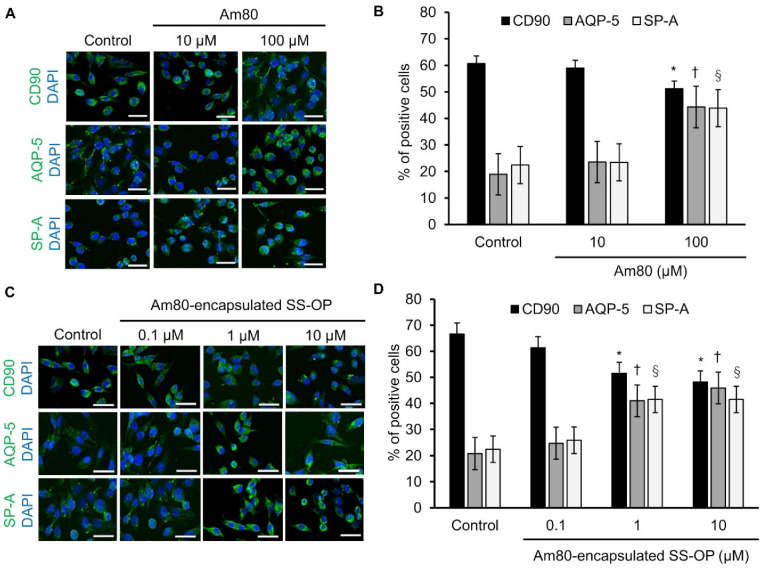
Am80-free or Am80-encapsulated SS-OP nanoparticles induced lung cell differentiation. Immunostaining of CD90, AQP-5, and SP-A after (**A**) the administration of free Am80 (10 and 100 μM) or (**C**) Am80 (0.1, 1, and 10 μM)-encapsulated SS-OP nanoparticles, which was observed by BZ9000, ×20, Scale Bar: 20 µm. The percentage of positively stained cells. The numbers of CD90-, AQP-5-, SP-A-, and DAPI-positive cells after treatment with (**B**) free Am80 and (**D**) Am80-encapsulated SS-OP nanoparticles. Each value represents the mean ± S.D. (n = 4–5). * *p* < 0.05 for CD90, ^†^ *p* < 0.05 for AQP-5, and ^§^ *p* < 0.05 for SP-A group comparisons, Dunnett’s test, vs. control group.

Free Am80 showed the differentiation induction effect in our previous study [21]. The differentiation inducibility of Am80 and Am80-encapsulated SS-OP nanoparticles was examined using Calu-6 cells, which are undifferentiated cells and were previously used in a cellular model of differentiation induction [33]. Calu-6 was exposed to 10 and 100 μM of Am80 and 0.1, 1, and 10 μM of SS-OP nanoparticles encapsulated with Am80. Then, cellular immunostaining was performed using the undifferentiated cell marker CD90, the type I alveolar epithelial cell marker AQP-5, and the type II alveolar epithelial cell marker SP-A, and observed using a BZ-9000. As a result of calculating the positive cell rate, a differentiation induction effect was observed at 100 μM in the Am80 exposure group (Figure 2A,B), while a differentiation induction effect was observed at 1 and 10 μM in the Am 80-encapsulated SS-OP nanoparticle exposure group (Figure 2C,D). From the above, it was clarified that the differentiation induction effect was observed at 1/100 of the concentration of the single substance by encapsulating Am80 in SS-OP nanoparticles. In our previous study, Am80 had a differentiation inducing ability in lung cells [21]. The present study showed that Am80 encapsulated in SS-OP nanoparticles also had a differentiation-inducing effect.

### 3.2. Alveolar Repair Effect and Tissue Immunostaining

#### 3.2.1. Evaluation by the Mean Linear Intercept (Lm)

**Figure 3:**
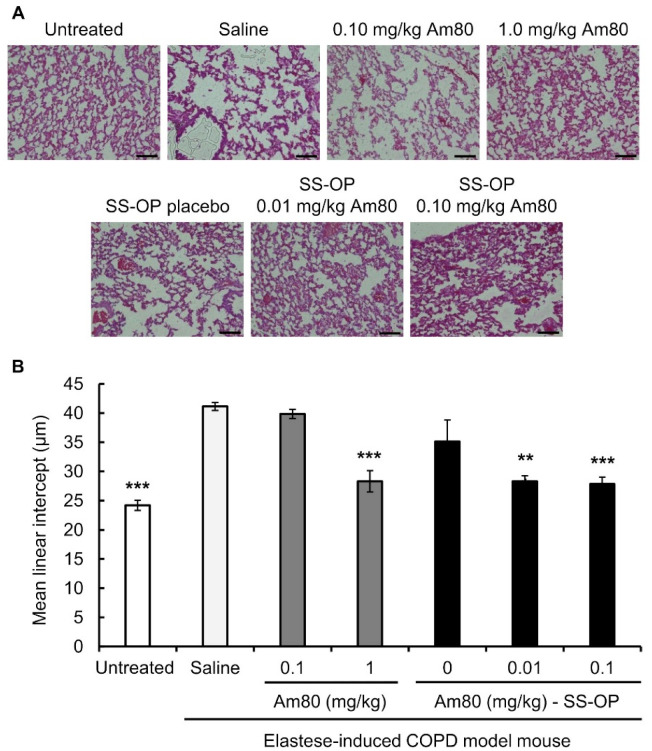
Effect of free Am80 or Am80-encapsulated SS-OP nanoparticles on Lm in the elastase-induced COPD model mouse. (**A**) A lung section observed under a BZ-9000 microscope (×20. Scale bar: 100 μm). (**B**) The mean linear intercept (Lm). Sections were stained with H&E. Data represent the mean ± S.E. (n = 5–8). ** *p* < 0.01 and *** *p* < 0.001, Dunnett’s test, vs. saline group.

The alveolar repair effect of SS-OP nanoparticles encapsulated with Am80 was investigated using the elastase-induced COPD model mouse. Elastase-induced COPD model mice were treated with free Am80 (0.10 or 1.0 mg/kg) or SS-OP nanoparticles containing Am80 (0.01 or 0.10 mg/kg) by pulmonary administration twice a week for 3 weeks. The untreated group was kept under the same conditions as the other groups for the same period without the administration of the drug solution. As a result, alveolar destruction was observed in the saline group, the 0.1 mg/kg free Am80 group, and the placebo group. On the other hand, in the 1.0 mg/kg free Am80 group and the 0.01 and 0.10 mg/kg Am80-encapsulated SS-OP nanoparticles group, the alveolar size was smaller in comparison to the saline group (Figure 3A).

The Lm value calculated from images of H&E-stained tissue was 41.13 ± 0.69 μm in the saline group and 39.84 ± 0.78 μm in the 0.10 mg/kg free Am80 group, while it was 28.29 ± 1.82 μm in the 1.0 mg/kg free Am80 group, which was similar to the value in the untreated group (24.18 ± 0.86 μm). On the other hand, the Lm of Placebo SS-OP nanoparticles was 35.1 ± 3.69 μm, while that of SS-OP nanoparticles containing 0.01 and 0.10 mg/kg of Am80 was 28.29 ± 0.98 μm and 27.83 ± 1.19 μm, respectively, which was similar to the Lm of the untreated group (Figure 3B).

#### 3.2.2. Tissue Immunostaining

**Figure 4:**
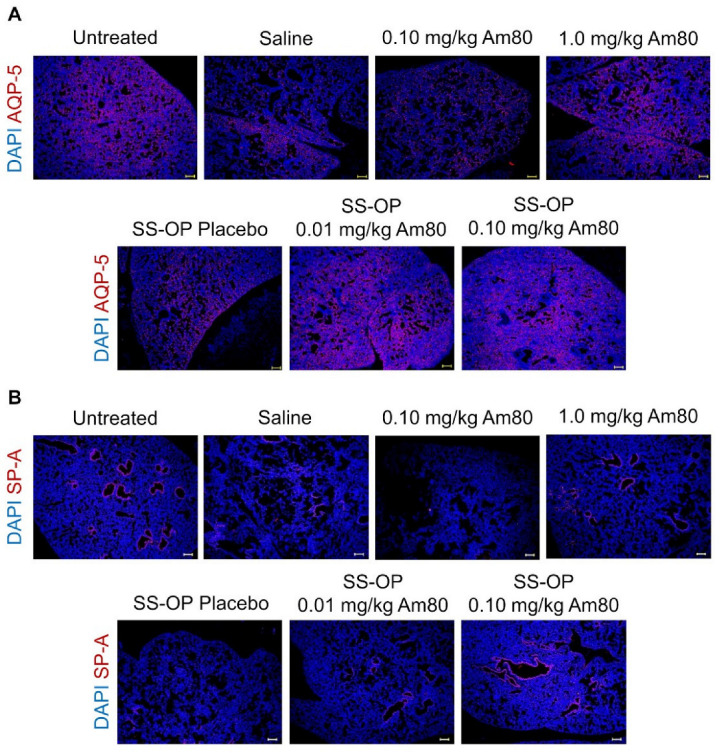
In vivo induction of differentiation by Am80-encapsulated SS-OP nanoparticles. Lung sections stained for (**A**) AQP-5 or (**B**) SP-A with Alexa Fluor 594, and DAPI nuclear staining observed using a BZ-9000 fluorescence microscope, ×4, Scale bar: 200 μm.

Tissue immunofluorescence staining was performed on lung tissue sections, and observation was performed using a BZ-9000. In the 0.10 mg/kg Am80 and placebo SS-OP nanoparticle groups, the level of AQP-5, a type I alveolar epithelial cell marker, did not increase in comparison to the saline group. On the other hand, the AQP-5 expression in the 1.0 mg/kg free Am80 group and the 0.01 and 0.1 mg/kg Am80-encapsulated SS-OP nanoparticles groups was similar to that in the untreated group (Figure 4A). A similar trend was observed in SP-A, a type II alveolar epithelial cells marker (Figure 4B).

### 3.3. Improvement of Respiratory Function

**Figure 5:**
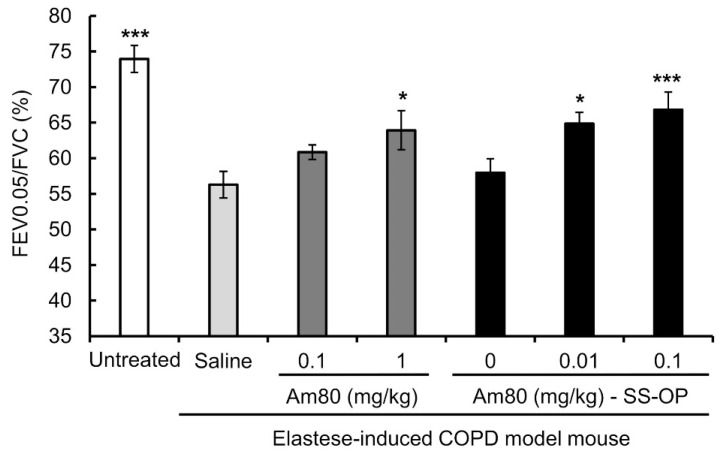
The respiratory-function-improving effect of free Am80 or Am80-encapsulated SS-OP nanoparticles in the elastase-induced COPD model mouse. FEV0.05/FVC was measured by flexiVent. Data represent the mean ± S.E. (n = 3–6). * *p* < 0.05 and *** *p* < 0.001, Dunnett’s test, vs. saline group.

**Figure 6:**
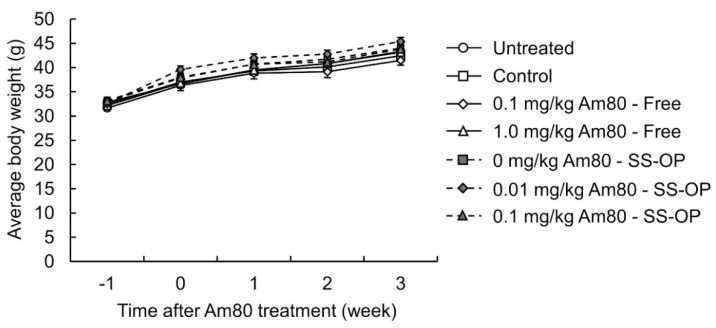
Effects of free Am80 and Am80-encapsulated SS-OP nanoparticles on the body weight decrease. Elastase-induced COPD model mice were treated with 0.1 and 1.0 mg/kg of free Am80 and 0.01 and 0.1 mg/kg of Am80-encapsulated SS-OP nanoparticles twice a week by pulmonary administration. The mean body weights measured at -1 (elastase administration point), 0, 1, 2, and 3 weeks after starting the administration of Am80 are shown. Data represent the mean ± S.E. (n = 5–7).

To investigate the effect of SS-OP nanoparticles containing Am80 on the improvement of the respiratory function, elastase-induced COPD model mice were treated with the drug solution, by pulmonary administration, twice a week for 3 weeks. The FEV0.05% was then measured using flexiVent. The FEV0.05% was 56.28 ± 1.88% in the saline group and 60.86 ± 1.02% in the 0.10 mg/kg free Am80 group, while that in the 1.0 mg/kg free Am80 group was 63.93 ± 2.73%; this demonstrated a significant improvement in the respiratory function in comparison to the saline group. On the other hand, in the placebo group, the FEV0.05% was 57.95 ± 1.99%, while the FEV0.05% in the 0.01 mg/kg and 0.10 mg/kg Am80-encapsulated SS-OP groups was 64.87 ± 1.57% and 66.81 ± 2.48%, respectively. This represented a significant improvement in respiratory function in comparison to the saline group (Figure 5). In addition, we measured body weight changes in untreated mice and elastase-induced COPD model mice that received free Am80 and Am80-encapsulated SS-OP nanoparticles in order to evaluate the systemic effect of pulmonary administered Am80 and SS-OP nanoparticles and found no significant changes in body weight (Figure 6, Table 2).

## 4. Discussion

As for emphysema treatment with retinoids, intraperitoneal (i.p.) administration of ATRA was performed in elastase emphysema model rats, and its therapeutic effect was reported [14]. However, no significant improvement was observed in human clinical trials with oral administration [17,18]. The effect of i.p. administration of ATRA on improving emphysema has been reported both in cases where a therapeutic effect was observed and not observed [14,35,36]. However, there are some points to discuss in the experimental conditions, such as the type of mice used, the route of administration of ATRA, and the method of administration. Other methods, such as oral and i.p. administration, fail to deliver drugs locally to the lung [33]. However, aerosolized ATRA exposed to the nose only did not improve emphysema [37]. On the other hand, an inhalation study of liposomal ATRA in humans showed an improvement in respiratory function and blood gases [38], suggesting that appropriate routes and methods of administration and the use of drug carriers may be effective treatments. In addition, ATRA metabolic enzymes are also present in the lungs [39,40]. Therefore, we investigated the usefulness of Am80 with higher metabolic stability than ATRA and reported that Am80 provided higher differentiation-inducing and alveolar repair effects than those of ATRA [21]. In this study, we investigated the differentiation-inducing effects and emphysema-improving effect of Am80-encapsulated SS-OP nanoparticles, which are expected to have more efficient drug transport, aimed to reduce the administration dose for the development of a powder inhalation formulation for clinical application. 

SS-OP nanoparticles with and without Am80 were prepared and their physical properties were evaluated. As a result, the average particle size of both nanoparticles was approximately 120–130 nm, with a unimodal particle size distribution and neutral zeta potential, and showed no significant difference between SS-OP nanoparticles with and without Am80 (Table 1). Accordingly, it was indicated that the encapsulation of Am80 had little effect on the physical properties.

In addition, as a result of examining the differentiation induction potency of Am80 and Am80-encapsulated SS-OP nanoparticles using the Calu-6 cell, which is an undifferentiated cell, the differentiation induction effect of Am80-encapsulated SS-OP nanoparticles, in comparison to Am80 alone, was shown at a concentration of 1/100. 

We evaluated the distribution of type I and type II alveolar epithelial cells in drug-treated elastase-induced COPD model mice. As a result, increases in Type I and Type II alveolar epithelial cell markers were observed in the groups treated with 1.0 mg/kg of Am80 alone and 0.01 mg/kg or 0.10 mg/kg of Am80-encapsulated SS-OP nanoparticles, in which alveolar repair effects were observed (Figure 3 and Figure 4). These results indicate that tissue repair with Am80 alone and Am80-encapsulated SS-OP nanoparticles is accompanied by an increase in type I and type II alveolar epithelial cells, which mainly comprise the alveoli.

The Lm was calculated from images of lung tissue sections. The Lm value reflects the mean spatial diameter and is used as an indicator of alveolar space expansion. Alveolar destruction is said to occur as the size of the alveolar air space increases [41]. In the present study, the Lm value of the 0.10 mg/kg Am80 did not differ from that in the saline group, while the value in the 1.0 mg/kg Am80 group showed a significant difference from that in the saline group, confirming alveolar repair. As in our previous study [21], 1.0 mg/kg of Am80 alone produced alveolar repair effects. In the case of SS-OP nanoparticles, when placebo particles were administered without Am80, the Lm value did not differ from that of the saline group, but when SS-OP nanoparticles with 0.01 mg/kg or 0.10 mg/kg of Am80 were administered, the improvement in Lm was significant and similar to that observed in the 1.0 mg/kg Am80 alone group. These results indicate that 0.01 mg/kg of Am80 can induce alveolar repair in SS-OP nanoparticles.

The respiratory function was measured in mice with elastase-induced COPD after the administration of Am80 alone or Am80-encapsulated SS-OP nanoparticles. As COPD is a respiratory disease with airflow obstruction, the FEV1.0% is measured in pulmonary function tests as an index of airflow obstruction [42]. Considering that the respiratory frequency of mice is higher than that of humans, the respiratory function was evaluated by measuring the FEV0.05%. As a result, it was found that 1.0 mg/kg of Am80 alone and 0.01 mg/kg of Am80 in SS-OP nanoparticles improved the respiratory function (Figure 5). In addition, an alveolar repair effect at the same dose was also demonstrated in Figure 4, suggesting that 1/100 of the dose of the Am80-free body can induce the histological and functional improvement of the pathophysiology of COPD by encapsulating Am80 in SS-OP nanoparticles. This result is consistent with the in vitro results, and the same effect as in vitro can be used to successfully reduce the dose to as much as 1/100.

The drug solution administered by our pulmonary administration method is distributed throughout the lung, including the alveolar region [33,34]. In addition, pulmonary administration of Am80 by our method to elastase-induced COPD model mice results in alveolar repair [21]. In this study, encapsulated Am80 showed an alveolar repair effect and differentiation-inducing effect in vivo (Figure 3, Figure 4 and Figure 5). This suggested that Am80-encapsulated SS-OP nanoparticles distributed in the alveolar region as well as free Am80. In addition, our previous study revealed that Am80 acts on alveolar epithelial progenitor cells and induces their differentiation into alveolar epithelial cells [21]. Am80-encapsulated SS-OP nanoparticles also showed a differentiation-inducing effect more effectively (Figure 3). This suggested that Am80 reached alveolar cells in in vivo experiments. SS-OP is incorporated into cells and efficiently escapes endosomes and releases the drug as an internal seal drug by efficiently self-degrading with the phenyl ester group of SS-OP [25]. It is reported that the site of action of Am80 is the nuclear receptor RAR [23,24]. Therefore, the results in this study suggested that Am80 may efficiently transfer into the cytoplasm and act on RAR, but this study did not show it. We will investigate the intracellular kinetics of SS-OP nanoparticles in the next study.

The effective dose in animal studies in mice is generally considered to be 12 times that in humans [22]. In this study, 0.01 mg/kg of Am80 was found to be effective for the treatment of COPD when encapsulated in SS-OP nanoparticles. Therefore, the effective dose in humans is estimated to be 0.05 mg/kg, based on an adult body weight of 60 kg. This value is similar in volume to the powder inhalation preparation that is currently used for COPD treatment in the clinical setting [43]. Therefore, the development of a clinically applicable inhalant formulation of Am80 by encapsulating Am80 in SS-OP is expected, and the development of a powder inhalation formulation of Am80-encapsulated SS-OP nanoparticles is currently being investigated.

## 5. Conclusions

In this study, Am80-encapsulated SS-OP nanoparticles showed a therapeutic effect in a mouse model of COPD at a dose that was 1/100 that of Am80; thus, the dose of Am80 was successfully reduced. From this result, the clinical dose was estimated to be 0.05 mg/60 kg, and it was suggested that a powder inhalation formulation could be applied in the clinical setting, and that Am80-encapsulated SS-OP nanoparticles might be useful as a curative drug for COPD.

## Figures and Tables

**Table 1:** Physicochemical properties of SS-OP nanoparticles.

Particle	Am80 Concentration (mM)	Am80-Encapsulated Percent (%)	Particle Size	P.I.	Zeta Potential (mV)
SS-OP (Placebo)	-	-	126.3 ± 2.32	0.07 ± 0.03	−8.63 ± 4.72
SS-OP (Am80)	1.78 ± 0.21	71.3 ± 8.49	125.7 ± 2.40	0.10 ± 0.02	−4.07 ± 1.54

Each value represents the mean ± S.D. (n = 3).

**Table 2:** Free Am80 and Am80-encapsulated SS-OP nanoparticles on the body weight decrease.

Group	Time after Am80 Treatment (Week)				
	−1	0	1	2	3
Untreated	32.8 ± 0.7	36.7 ± 0.9	39.5 ± 1.1	40.8 ± 1.2	43.3 ± 1.2
Control	32.4 ± 0.4	37.0 ± 0.9	39.3 ± 0.9	40.1 ± 0.9	42.4 ± 1.0
0.1 mg/kg Am80 -Free	31.7 ± 0.6	36.3 ± 1.1	38.8 ± 1.2	39.2 ± 1.2	41.5 ± 1.0
1.0 mg/kg Am80 -Free	32.2 ± 0.5	36.8 ± 0.7	39.5 ± 0.8	40.8 ± 0.5	43.2 ± 1.0
0 mg/kg Am80 -SS-OP	33.0 ± 0.4	37.9 ± 0.6	40.7 ± 0.8	41.7 ± 0.7	44.0 ± 0.8
0.01 mg/kg Am80 -SS-OP	32.6 ± 0.5	39.6 ± 0.7	42.0 ± 0.8	42.8 ± 0.8	45.4 ± 0.8
0.1 mg/kg Am80 -SS-OP	33.2 ± 0.7	38.0 ± 0.7	40.7 ± 0.7	41.2 ± 0.8	43.8 ± 0.7

Each value represents the mean ± S.E. (n = 5–7).

## Data Availability

Not applicable.
